# The phytomicrobiome: solving plant stress tolerance under climate change

**DOI:** 10.3389/fpls.2023.1219366

**Published:** 2023-09-07

**Authors:** Abdul Latif Khan

**Affiliations:** Department of Engineering Technology, University of Houston, Houston, TX, United States

**Keywords:** phytomicrobiome, plant growth, extreme environment, abiotic stress, metagenome

## Abstract

With extraordinary global climate changes, increased episodes of extreme conditions result in continuous but complex interaction of environmental variables with plant life. Exploring natural phytomicrobiome species can provide a crucial resource of beneficial microbes that can improve plant growth and productivity through nutrient uptake, secondary metabolite production, and resistance against pathogenicity and abiotic stresses. The phytomicrobiome composition, diversity, and function strongly depend on the plant’s genotype and climatic conditions. Currently, most studies have focused on elucidating microbial community abundance and diversity in the phytomicrobiome, covering bacterial communities. However, least is known about understanding the holistic phytomicrobiome composition and how they interact and function in stress conditions. This review identifies several gaps and essential questions that could enhance understanding of the complex interaction of microbiome, plant, and climate change. Utilizing eco-friendly approaches of naturally occurring synthetic microbial communities that enhance plant stress tolerance and leave fewer carbon-foot prints has been emphasized. However, understanding the mechanisms involved in stress signaling and responses by phytomicrobiome species under spatial and temporal climate changes is extremely important. Furthermore, the bacterial and fungal biome have been studied extensively, but the holistic interactome with archaea, viruses, oomycetes, protozoa, algae, and nematodes has seldom been studied. The inter-kingdom diversity, function, and potential role in improving environmental stress responses of plants are considerably important. In addition, much remains to be understood across organismal and ecosystem-level responses under dynamic and complex climate change conditions.

## Introduction

According to the Intergovernmental Panel on Climate Change (IPCC; https://www.ipcc.ch/), the accumulation of atmospheric CO_2_ entraps solar radiations, which can then be emitted back to the earth’s surface – increasing the global temperature. This, in turn, leads to the development of a pattern of climate modification termed the Global Climate Change (Abbass et al., 2022). Climate changes due to greenhouse gas (GHG) emissions have influenced the soil systems, natural plant productivity, and health (Koneswaran and Nierenberg, 2008). The increased atmospheric CO_2_ is due to extensive industrialization, urbanization, and natural resource use patterns, drastically creating an imbalanced environmental system (Zandalinas et al., 2021). CO_2_ levels have risen by 40% (~414.72 parts per million concentrations) – higher than in the pre-industrial era (Dlugokencky et al., 2018). These changes have influenced global rainfall, temperature patterns, and variable soil chemistry, considerably impacting the associated natural bioresources (plants and microbes) across terrestrial ecosystems. Climate-based changes, such as increasing or decreasing temperature and lack or over-abundance of water, can change the soil nutrients and essential chemicals, creating an imbalance in the ecosystem’s cycling system (Van Den Heuvel et al., 2020). It has been estimated that an increase of 3°C to 4°C would cause a reduction in plant productivity by 15 to 35% by the end of the 21^st^ century (Tayade et al., 2018). Other abiotic stresses (flooding, salinity, and heavy metals) have been estimated to reduce plant productivity by 51–82% (Cooke and Leishman, 2016). These changes have hindered the desired natural productivity of plants and their responses to combating abrupt climate changes. Also, this has threatened food security and human use values for future human generations.

Plants respond to external environmental stimuli by changing their biochemical and physiological relationship. Since microbes have been associated with plants throughout their life cycle, therefore, any small developmental or metabolic change also influences them. Microorganisms are the silent wheel that functions as the cradle of plant growth, stress signaling, and responses in terrestrial ecosystems. The microbes’ composition, structure, and richness are variable across different environmental systems and associations with host plants. These microbes live as endophytic (inside) or epiphytic (outside) modes of life with mutualistic, commensal, or parasitic relationships. “Microbiome refers to the total genetic material of microbial communities associated with plants in either of these modes and associations” (Hassani et al., 2018), whereas ‘holobiome’ or ‘holobionts’ is a sum of genomic material of host and associated biota – including all prokaryotic and eukaryotic organisms (Hassani et al., 2018; Lyu et al., 2021). A newer concept of ‘eco-holobionts’ argues exponentially regarding the ecological or ecosystem-based interaction of microbiome to identify plant-soil-animal-environmental functionalities (Singh et al., 2020; Wani et al., 2022).

**Figure 1:**
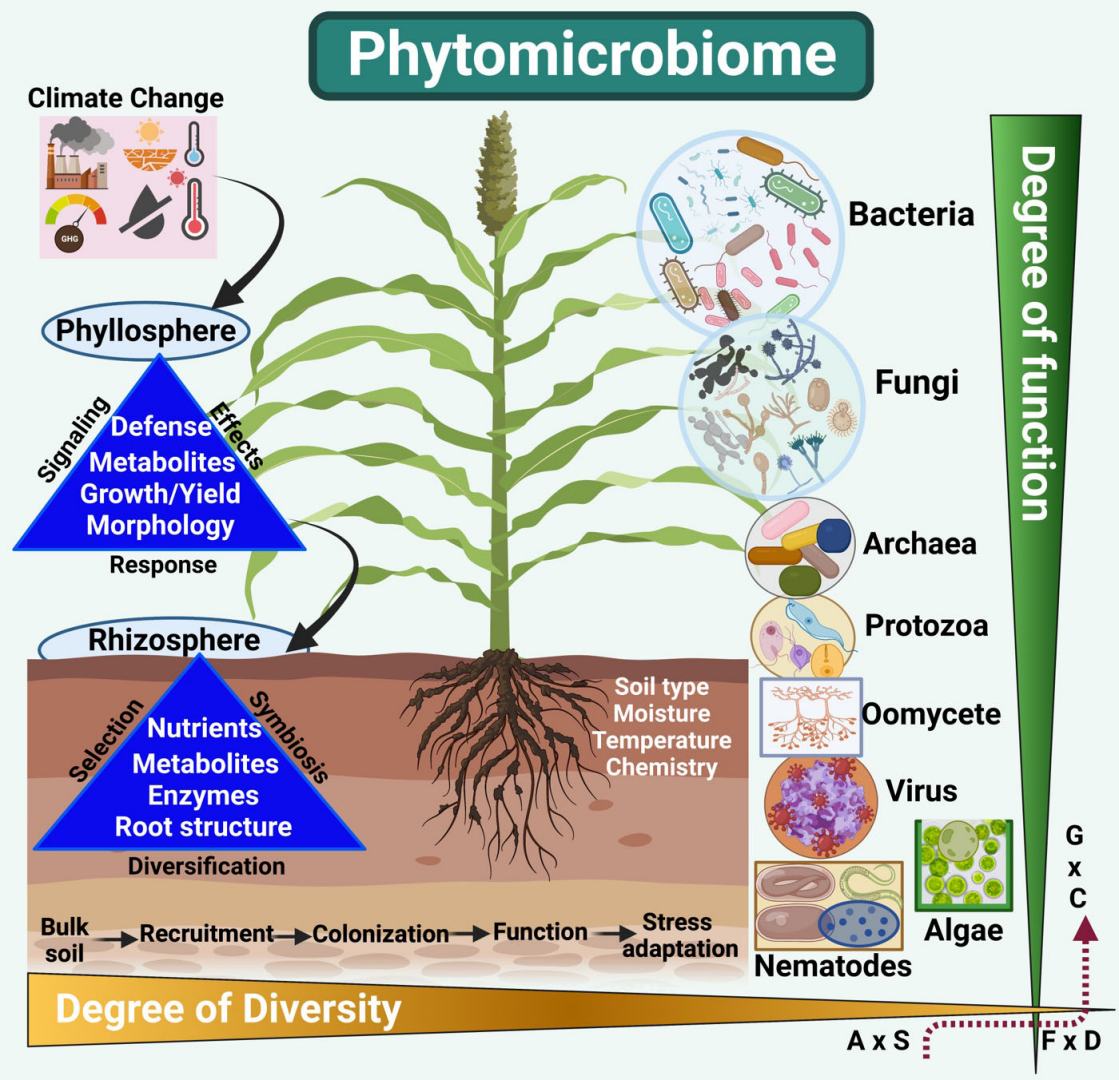
Climate change due to the emission of GHGs and resulting global temperature and rainfall patterns significantly impacts the plant’s photosynthesis, defenses, and yields. This drastically impacts soil health, microbial activities, nutrient mobilization, and uptake and secretion of signaling metabolites. Thus, impacting both the phyllosphere and rhizosphere parts of the plant life. Phytomicrobiome members (bacteria, fungi, protozoa, oomycetes, viruses, algae, and nematodes), on the other hand, drastically increase or decrease to respond to the change climatic changes (drought, heat, cold, flooding, salinity, etc.). The structure, diversity, and function significantly shift from higher to low or low to higher for specific phytomicrobiome players. For example, diversity can reduce from bacteria to viral species in a given phyllosphere and rhizosphere segment during abiotic stress. A similar perspective has been considered for the degree of function and diversity from nematodes to algae in soil systems alongside bacterial and fungal species during stress. The interactome of Abundance (A) *vs*. stress protection (S), function (F) *vs*. diversity (D), and genotype (G) *vs.* environment (condition C) is extremely complex and dynamic. Thus, the lack or abundance of a specific class of phytomicrobiome players can significantly impact a plant’s function and response to climatic stresses. (Created with BioRender.com).

More recently, the phytomicrobiome has been considered a “second functional genome” in addition to the host plant’s genome (Leach et al., 2017). A phytomicrobiome is a total sum of all microorganisms that successively develop relationships with plants during their growth stages. Although bacterial and fungal biomes have been studied extensively, some of the missing links of holistic interactions of archaea, viruses, oomycetes, protozoa, algae, and nematodes have seldom been studied together for their functional roles (**Figure 1**). The phytomicrobiome can provide a sustainable climate-smart plant growth and production solution to enhance abiotic stress tolerance. However, the responses of the phytomicrobiome depend upon the plant’s genotype and ability to cope with stress factors (Trivedi et al., 2022). Microbiome diversity and abundance have been significantly correlated with plants’ ability to grow in a specific environmental system (Leach et al., 2017; Trivedi et al., 2020). However, more basic knowledge is required to understand this dynamic and complex plant-biotic interaction. For example, there have been more than ~5,000 reports related to the human microbiome till the year 2022. Comparatively, in the case of plant sciences, these are very low, i.e., above 800 studies in SciFinder.

Phytomicrobiome envelopes a diversity of disciplines encompassing biotechnology, genomics, microbiology, plant physiology, food sciences, agriculture, bioeconomy, informatics, and medical sciences. Though there is a sharp increase in utilizing a holistic microbiome approach, the concept and required skills continuously evolve. Due to the advances in sequencing technologies and machine learning methods, a significant shift has been noticed from amplicon-based community analysis to in-depth molecular processes. Strategies such as metagenome-assembled - genomes, genome–resolved - metagenomics, genome-wide association, meta-transcriptomics, and genome editing for synthetic communities have recently gained attention (Khan et al., 2020; Trivedi et al., 2020; Chouhan et al., 2021). However, more needs to be understood across different ecological and environmental dynamics and changing global climates (**Figure 1**). To increase plant growth and production while reducing the environmental impact of the whole process, sustainable utilization of phytomicrobiome diversity can be an essential part of achieving stress tolerance (Chawade et al., 2018; Afridi et al., 2022).

## Phytomicrobiome diversity and function

The earth’s microbial diversity and richness have been estimated as ~1 trillion (10^12^) species distributed in 30 orders (Locey and Lennon, 2016; Thompson et al., 2017; Thaler, 2021). Contrarily, the Earth Microbiome Project has predicted that microbial diversity can be nearly 10 million species globally. The very least percentage of microbial diversity or function is known in both cases. The same is true for the availability of genomic sequence and culture stocks. Hence, a greater need exists to explore unique phytomicrobiomes and identify keystone species of extreme environments for potential agricultural benefits. The phytomicrobiome is an essential aspect of plant life where a continuous interaction of neutral-microbiomics (microbe with least functional role), patho-microbiomics (pathogens with antagonistic role), and core-microbiomics (functional microbiome) happens in the context of spatial or heterogeneous richness (Khan et al., 2020; Trivedi et al., 2020). The core microbiome is a significantly abundant microbial taxonomy in a given habitat. It performs a multi-factorial function, including plant growth promotion, abiotic stress controls, and defense against pathogens and pests in a robust manner (Xiong et al., 2020; Jiang et al., 2022).

In phytomicrobiome settings, microbes can range from bacteria, fungi, archaea, protozoa, oomycetes, viruses, nematodes, and algae. The degree of diversity (alpha–community scale and beta–between species) of the microbiome is important for plant growth (Pang et al., 2021; Andermann et al., 2022). The microbial function (production of metabolites and enzymes, nutrient mobilization and uptake, reproduction, and metabolic activities) are delicately interwind and complex in the phytomicrobiome setup (Trivedi et al., 2022). The diversity and function go side by side and vary significantly in a typical environmental setting (**Figure 1**). For example, the rhizosphere compartment will possess a higher diversity of microbes than the phyllosphere. Similarly, a significant variation in diversity and function has been proposed from bulk soil into rhizospheric soil and then root parts. The bulk soil provides a seed bank for plant expansion, selection, and recruitment of microbial diversity. Conversely, the phyllosphere (stem, secondary shoots, leaf, flowers, and seeds or fruits) has been the least studied. The recent literature suggests that microbial abundance sharply reduces from rhizosphere to phyllosphere (Pantigoso et al., 2022). This abundance can also be dependent on the host genotype and growth stages. The environmental settings can drastically impact diversity and function. All the abiotic factors (temperature, water, light, pH, etc.) dramatically impact microbiome species’ recruitment and colonization patterns. Thus, any abiotic stress factor, either long or short-term, low to severe, is directly proportional to phytomicrobiome structure. Also, the broad spectrum interactome of the phytomicrobiome with phytobiome has been studied in crop segregation.

The plant growing in extreme environmental conditions (xerophytes, halophytes, etc.) hosts a huge diversity of phytomicrobiome species. Exploring extreme and unique phytomicrobiome provides a pivotal resource for beneficial naturally competent microbes that can help to improve crop growth, productivity, and resistance against pathogenicity and abiotic stresses (D’hondt et al., 2021; Lyu et al., 2021; Ali et al., 2023). Several recent studies have shown that microbiome diversity and function (**Figure 1**) are affected by the following:

i. Short or long-term abiotic affecters like temperature, water (rainy or dry), soil chemistry, and nutrients cycling,
ii. Host’s type, developmental stage, and abilities of plants to establish successful symbioses with the core microbiome,
iii. Biotic affecters, such as the interactions of the core with hub microbiota and keystone species or interactions with pathogenic or commensals
iv. Soil size, type and surface, water, pH, and composition of macro and micro-nutrients in the rhizosphere
v. Presence or absence of essential exudates (primary, secondary, or specialized metabolites), enzymes (extracellular), and substrates for the growth and reproduction

Plants with a healthy phytomicrobiome provide a healthy soil system that can better sequester several beneficial nutrients and moisture compared with a poorly composed soil system (**Figure 1**). This can broadly impact plant biomass production, yield, and essential photosynthetic processes. A healthy phytomicrobiome also offers higher resilience to climatic stresses through various metabolites and enzymatic secretion in soil systems (Pang et al., 2021). Indeed, the agri-microbiome is gradually progressing in research; however, the phytomicrobiome and its niche in extreme ecosystems have been the least explored (Pfeiffer et al., 2017). Previous studies have evaluated the major players in a microbiome, especially the bacterial biome from different soil systems; however, little is known regarding exploring the depth of the cumulative phytomicrobiome, populations, and function in improving a crop’s resistance to stress (Mandakovic et al., 2018; Araya et al., 2020; Astorga-Eló et al., 2020; Khan et al., 2020). Exploring a unique trove of natural resources distributed across unique ecosystems is necessary to create more base knowledge and potential microbes for abiotic stress tolerance (Khanna et al., 2022). Increasing our mechanistic understanding and real-world experience of microbiome-plant interactions under drought, salinity, and heat stresses offers enormous potential for increasing the resilience of crops in such conditions (De Vries et al., 2020). Looking at the current focus on plant-microbe interaction, we also need to harness the stress tolerance mechanisms to improve plant growth in extreme conditions and focus on increasing plant yields.

We propose that utilizing naturally growing plants in extreme environments could be a vital resource of phytomicrobiome that can offer prospective benefits to crop plants during extreme plant growth conditions. For example, desert conditions cover over 30% of the earth, and plant and microbial life are confronted with extreme living conditions that depend significantly on scarce water and nutrients from the soil. Xerophytic succulent plants are the key players well-tailored to continuous episodes of abiotic stresses (drought, heat, and salinity) (Ndour et al., 2020; Peguero-Pina et al., 2020; Zeng et al., 2021). These extreme plants, due to their peculiar anatomy, withstand severe stress and are often unique in their (i) genetic makeup, (ii) physio-photosynthetic responses, (iii) essential metabolites production, and (iv) core microbial symbiosis (Griffiths and Males, 2017; Heyduk, 2021). The symbiotic microbiota (bacteria and fungi) in the root (rhizosphere) and shoot regions (phyllosphere) have been recently proposed for their potential role in improving host life and fitness (Khan et al., 2020; Trivedi et al., 2020; Sharifi et al., 2022).

For example, the drought-promoting microbiome in desert farming improved overall photosynthesis and plant biomass by 40% (Marasco et al., 2012). Several studies are reporting the microbiomes of the Atacama desert (Araya et al., 2020; Contador et al., 2020; Menéndez-Serra et al., 2020), Lejía Lake (Mandakovic et al., 2018), empty quarters Oman (Khan et al., 2020), Sonoran desert (Andrew et al., 2012; Finkel et al., 2012; Gornish et al., 2020), Mojave Desert (Pombubpa et al., 2020), saline lakes (Monegros Desert, Spain) (Menéndez-Serra et al., 2020), the atmospheric microbiome in the Eastern Mediterranean (Mazar et al., 2016), and the seed-associated microbiome from Southern Chihuahuan Desert (Menéndez-Serra et al., 2020). Some of the succulent and arid land plant species recently analyzed for their microbiome are the *Agave* species (Flores-Núñez et al., 2020), *Aloe vera* (Akinsanya et al., 2015), *cacti* (Fonseca-García et al., 2016), pineapple (Putrie et al., 2020), Aizoaceae (Pieterse et al., 2018), frankincense-producing tree (*Boswellia sacra*) (Khan et al., 2016a; Khan et al., 2017). These studies showed highly diverse rhizosphere colonization with *Actinobacteria, Proteobacteria*, *Firmicutes, Actinobacteria, Acidobacteria*, and *Bacteroidetes* (Citlali et al., 2018; Flores-Núñez et al., 2020). These reports suggest that microbial symbionts of these plants provide stress-protecting benefits that could be replicated in agroecological settings. For example, culturable microbial strains such as *Preussia* sp. BSL10 (Khan et al., 2016b) and *Sphingomonas* sp LK11 (Khan et al., 2014) were isolated from xerophytic plants and were able to produce beneficial metabolites (gibberellic acid and auxins). The inoculation in mono-culture semi-sterile conditions significantly improved plant growth and biomass. Currently, most studies have mentioned microbial communities’ (fungi and bacteria) distribution, abundance, and diversity, where the role of other microbiome players has been least identified (Finkel et al., 2017). Below are some of the critical questions that still need to be answered:

**Q1:** What core/hub/key phytomicrobiome species are consistently present with plants exposed to extreme climate changes?
**Q2:** How does a single or consortium of core-microbiome species benefit a plant’s life?
**Q3:** What gene networks and biosynthetic pathways contribute to plants’ survivability in stressed environments?
**Q4:** How does the phytomicrobiome help the host on a prolonged and short-term basis?
**Q5:** How to replicate the essential benefits of core microbiome into crop’s abiotic stress resistance from germination to yield levels?
**Q6:** What molecular and metabolic networks assist successful and long-term symbiotic relationships during climate change with crops?

**Figure 2:**
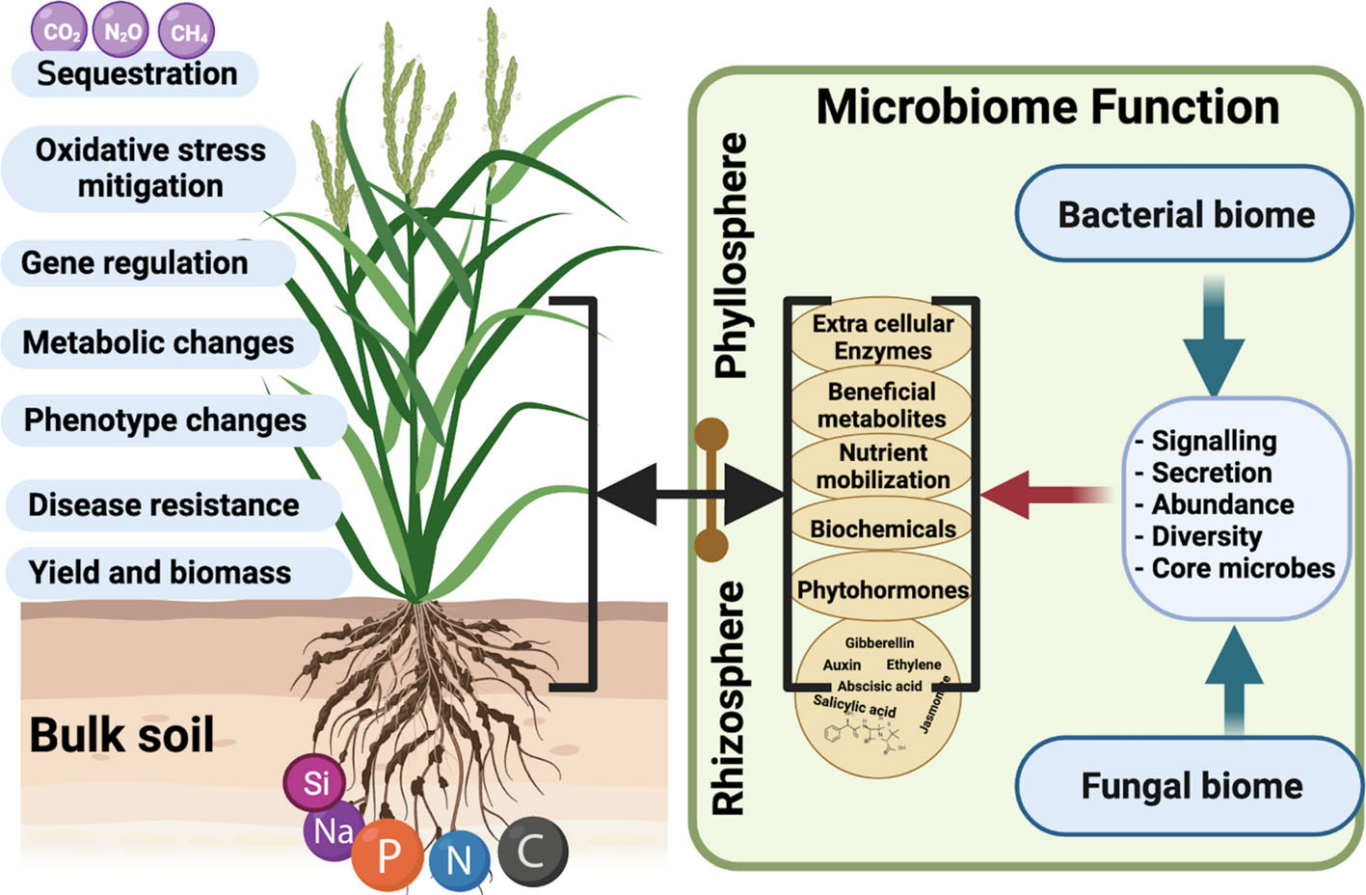
The Phytomicrobiome responds to host plant growth and stress tolerance by producing several signaling molecules. These secretomes directly influence plant microbiome structure and diversity. Hence, each climate-induced stress factor would directly challenge the composition and function of core-microbiome species associated with a host. Microbiome members’ associated plant growth and stress aversion defenses impact critical aspects of plant life (growth, metabolism, resistance to stress, gene regulation, and biomass yield). This also impacts nutrient cycling, transport, mobilization, and translocation inside plant tissues during optimal or stressful conditions. The core-microbiome function drastically changes and shifts from the rhizosphere into the phyllosphere. (Created with BioRender.com).

Although the key questions of a microbiome study and environmental evaluation are well-defined, there is a dire need to advance knowledge on above questions. The recent microbiome literature, focuses more on the (i) “What is there”? and (ii) “What are they doing”? However, (iii) “what can they do” has been frequently overlooked (Khan et al., 2020; Trivedi et al., 2020) (**Figure 2**).

## Specialized metabolites production by phytomicrobiome – a trait to counter stress tolerance

The phytomicrobiome players can help in nitrogen fixation, soil carbon, and phosphorus cycling to improve root growth and development (Kusari et al., 2012; Backer et al., 2018). The majority of current literature shows that microbes have been known to improve plant growth by (i) nutrient solubilization and uptake, (ii) enzymes secretion, (iii) secondary metabolites, and (iv) phytohormones production (**Figure 2**) (Pang et al., 2021). The biochemical substance secretion and molecular signaling mechanisms adopted by microbiome functioning play a key role in host-stress responses. For example, phytohormones are signaling molecules and chemical messengers that play an essential role in plant growth and development (Hemelíková et al., 2021). Phytohormones produced by microbes include gibberellins (GA), auxin, cytokinin, salicylic acid, abscisic acid, etc. Most GA-producing fungi belong to *Ascomycetes* and *Basidiomycetes* (Takeda et al., 2015; Salazar-Cerezo et al., 2018; Sharifi et al., 2022) and have been identified as plant growth promoters during stress conditions. Most of these studies are based on mono-culture conditions, and least is known on their role in mix-community structures.

There are few examples of bacterial strains known for GA production, whereas auxin production is more bacterial trait than fungal. Still, some reports show GA production and related genes in bacterial strains (Nett et al., 2017; Lemke et al., 2019). Despite some major fungal species, the biosynthetic pathway of GA has yet to be fully explained in both bacterial and fungal strains. Auxins, on the other hand, are more known for bacterial production than fungal and have been well explained for their biosynthetic gene clusters. Unfortunately, there is a significant knowledge gap regarding microbes’ axenic *vs*. holoxenic phytohormonal production abilities and their function in mitigating climatic stress factors. The prospect of such beneficial strain is exceptionally high for plant responses to climate change. For example, biopriming of maize seeds with GA-producing bacterial strains showed markedly enhanced maize seedling tolerance to oxidative stress. This also improved drought tolerance by up to 20% (Shaffique et al., 2022). When the rhizobacterium *Azospirillum brasilense* was introduced to the roots of Arabidopsis, the host plant displayed increased endogenous abscisic acid (ABA) levels and drought tolerance (Eichmann et al., 2021). Other reports suggested that GA producers significantly reduced the ABA level during drought, heat, and heavy metal stresses – indicating microbial role in reprograming the immune stress responses (Khan et al., 2015; Khan et al., 2020).

In the case of enzymes, one noteworthy exception is 1-aminocyclopropane-1-carboxylate (ACC) deaminase, a bacterial enzyme that helps maintain root growth by keeping a check on the ethylene level (Ali et al., 2014). In stress conditions, plant activates ACC synthase and or oxidase to increase ethylene production, which can lead to reducing root growth activities – causing declining plant production. In such conditions, the symbiotic microbiome can produce ACC deaminase that helps down-regulate ethylene levels, assisting the plant in escaping or minimizing stress conditions (Jha et al., 2021). The ACCd activities can help improve root colonization and combat pathogenic infections. Another potentially important mechanism is the physical sheathing of the root by either bacteria or mycorrhizae, protecting it from water loss. Such a mechanism, which requires the establishment of dense biomass on the root, necessitates compatibility with the plant’s immune system. Effective biofilm formation on roots also strongly depends on synergistic interactions among multiple microbial taxa (Berendsen et al., 2018). Thus, consortia of single or multiple genera can drastically reduce the negative impacts of stress–providing plant growth-promoting effects (Finkel et al., 2020; Fitzpatrick et al., 2020; Salvato et al., 2022). The inoculation of these fungal and bacterial strains can improve plant biomass and stress resistance by modulating antioxidant enzymes and growth-related gene expression. Contrarily, the functions of phytohormone-producing microbiome players in a consortium have been least known. How microbial symbiosis and community structures intervene in the signal-to-response potentials has not been elucidated yet.

## Elucidating phytomicrobiome for plant responses to climate change

According to comparative metatranscriptomics, the active rhizosphere microbiome of wheat, oat, and pea has revealed kingdom-level variations and functions (Turner et al., 2013). The sorghum root-associated microbiome demonstrates enhanced transcriptional activity of genes involved in glucose and amino acid metabolism and transport in response to drought stress, primarily due to changes in actinobacterial activity and function (Xu et al., 2018). In soybean, the *Bradyrhizodium* and *Gammaproteobacteria* (*Proteobacteria* phylum) were dominant and associated with crop productivity during abiotic stresses (Chang et al., 2017). Similarly, the *Actinobacteria, Chloroflexi*, *Proteobacteria* and *Ascomycota*, *Basidiomycota*, and *Mortierellomycota* phyla were significantly dominant in the soybean that was grown in different soil textures (Trépanier (2019). *Firmicutes* are known to have anaerobic species, which is most likely why they play a significant role during flooding stress (Martínez-Arias et al., 2022). Contrarily, the *Proteobacteria* are more abundant in flooding with elevated CO_2_, which is known to play a crucial role in abiotic stress environments (Vaishnav et al., 2018). Recent studies have shown that taxa from a single genus or family in the rhizosphere or phyllosphere of rice and *Arabidopsis* plants offer increased drought stress tolerance (Finkel et al., 2020). Furthermore, microbiome-mediated temperature tolerance has been reported for maize (Tiziani et al., 2022), rice (Liu et al., 2023), wheat (Chen et al., 2022), and *Arabidopsis* (He et al., 2022). In the endospheric microbiome, the inoculation with endophytic bacteria showed upregulation of cold stress tolerance-related genes (Theocharis et al., 2012). Single species of bacterial endophytes are reported for the accumulation of cold stress-linked metabolites such as essential sugars (starch), amino acids (proline), and phenolic (catechol) compounds in plant tissues (Ayilara et al., 2023) Microbial communities help soybean to solubilize silicon, phosphorus and produce phytohormones and organic acids (Kang et al., 2017). The microbe-mediated plant growth and stress tolerance of individual microbial taxa have been long known, whereas how the endospheric microbiome offers tolerance has yet to be fully understood.

To identify the underlying mechanisms of microbiome-mediated plant growth and stress tolerance, metagenomic and metatranscriptomic profiling are used to discover the function and metabolic pathways used during plant-microbe-stress interactions. Such interactions and tools have recently helped build microbial communities as drought stress biosensors were a recent breakthrough (Zolti et al., 2020). Despite being a valuable tool for understanding the roles of active members of plant-associated microbiomes, poor correlations between transcription and translation need the development of proteomic and metabolomic approaches to supplement transcriptomics. Metaproteomic studies of microbial communities from the rhizosphere (Bona et al., 2019; Sharifi et al., 2022) and phyllosphere of agricultural plants have provided direct insights into their molecular phenotypes. The leading members of the microbiome and the proteins found in distinct plant-associated settings have shown remarkable stability in this limited research (Knief et al., 2012; Bona et al., 2019).

Although metabolomic methods are rapidly being employed to diagnose plant diseases and their etiological agents, their utility in microbiome science still needs to be improved (Adeniji and Babalola, 2020). According to early research, the rhizosphere microbiome alters the phyllosphere metabolome, and these alterations are linked to differences in insect feeding behavior (Badri et al., 2013; Pantigoso et al., 2022). Changes in root metabolome can shape specialized microbial populations, affecting plant performance and plant-herbivore interactions in the future (Huang et al., 2019). Small compounds from microbes (organic acids, amino acids, sugars, volatiles) and plant exudates (flavonoids, phenolics, terpenoids, phytohormones) that drive plant–microbiome communications and interactions require metabolome information to be detected and quantified (Trivedi et al., 2020). Multi-omics (metabolome, ionome, microbiome, and phenome) and integrated informatics were recently applied in an agroecosystem to uncover intricate connections between plant characteristics, metabolites, microbes, and minerals (Ichihashi et al., 2020). We believe that better sample preparation (e.g., removal of host sequences for shotgun sequencing and transcriptomics, as well as universal protein extraction for proteomics), more datasets in the publicly available databases, and the development of algorithms and computational tools for data integration will allow multi-omics approaches to unlock the genotype-phenotype spectrum in an agricultural setting to their full potential. This will also help combat climatic change’s impacts on crop production.

## Native synthetic communities and plant growth promotion during stress

The phytomicrobiome relies more on amplicon and metagenome sequencing and data analytic approaches. However, in recent years metagenomics coupled with the culture-dependent synthetic communities (SynComs) have arguably provided more mechanistic insights (Ke et al., 2021). This is based on isolating and identifying large-scale reconstruction of bacterial and fungal cultures as SynCom. Recently both bottom-up and top-down approaches have been proposed for identifying SynCom functions (San León and Nogales, 2022). Such SynCom is screened via high throughput for potential functions during climate-induced stresses. Recent studies show that such approaches are highly beneficial in comparing stressed and non-stressed phytomicrobiome functions. Several recent examples illustrate the true potential of several PGPs as SynCom. Many PGPs have been isolated and identified as biofertilizers, biostimulants, and biocontrol agents. Both native or non-native culturable SynCom and genetically modified microbes have been extensively researched and used by Synlogic, Pivot Bio, JOYN Bio, NOVOME Biotechnologies, 64-X, etc. However, applying SynCom microbes to fields for commercial adoption has been a challenge until now. This is likely because the more-resilient existing microbial communities exclude the new microbes (De Vries et al., 2018). One of the recent reviews focuses on developing new microbes to sustainably support plant health, defense, and productivity by understanding and isolating core-microbiome species **(** Ke et al., 2021 **).** Also, their large-scale encapsulation and potential to be utilized as climate stress-protective microbial communities have yet to be fully understood. Knowledge derived from these studies may provide strategies for using plant growth-promoting microbes in fields. Very soon, rhizospheric and phyllosphere microbiome engineering strategies will be adopted to increase sustainable agricultural production (Backer et al., 2018), specifically climate-smart agriculture.

Similarly, to resolve these challenges, microbiome engineering based on synthetic biology is catching the attention rapidly as a new approach to developing synthetic microbial communities (modified SynComs) (Kaminsky et al., 2019). Modified SynComs are consortia of microbes synthetically designed to mimic the observed function and structure with the natural microbiome. The aim is to minimize the complexity of the community while retaining the biotic and abiotic interactions of the host and microbes (Chouhan et al., 2021). The SynComs are potentially delivered to specified locations or organs of a plant at different growth stages by environmental conditions. However, genetically modified microorganisms are monitored strictly (Saad et al., 2020; Ke et al., 2021). In near future, an engineered or synthetic microbiome will be a safe and sustainable approach toward sustainable agriculture (Ke et al., 2021). However, several biosafety, ethical and environmental issues, and hazards must be considered. Attention should be paid to not impacting an ecosystem’s natural *vs*. synthetic microbiome structure.

Thus, SynCom is a valuable approach to manipulating and understanding natural communities. SynCom is also used to identify plant genetic factors determining the assembly of the leaf or root‐associated microbial communities. But few studies have examined the role of agroecological interactions of SynCom colonization. Microbial inoculants are increasingly considered an effective complementary tool in the context of agroecosystem sustainability and productivity (Liu et al., 2022) However, the mechanisms that underpin positive impacts on plant fitness remain poorly understood, constraining the development and adoption of effective SynCom. Considering global temperature changes, using beneficial SynCom can be an ideal strategy to overcome the challenges of a sustainable agri-ecosystem. This can be achieved through developing knowledge of phytomicrobiome networks via multi-OMIC methods (Khan et al., 2015; Khan et al., 2020; Trivedi et al., 2020). Thus, affecting the function of a single microbe applied to a plant field (De Vries et al., 2018). Hence, in the current era, instead “one-microbe-at-a-time” approach, alternative SynComs can indicate a better prospect of function to survive the agri-ecosystem environment (Zhuang et al., 2021). Although this field is still progressing, one can still ask to what extent SynCom derived from a natural microbiome effectively improves plant growth, especially with the inclusion of abiotic stress factors (La Vega-Camarillo et al., 2023). The current literature shows a significant need to harness stress tolerance mechanisms to improve plant biomass in extreme conditions (Van Der Heijden and Hartmann, 2016; De Vries et al., 2020). The concurrent molecular signaling and the role of physio-genomic level responses of plants have been seldom studied in the context of climate stresses and this also true of their microbiome functions.

## Conclusion and future prospective

In conclusion, the phytomicrobiome (either core, satellite, or key), its dynamics in changing climatic conditions, and potential regulators from extreme terrestrial environments can benefit the crop production system. The phytomicrobiome knowledge can be extended to (i) understand wild and cultivated microbiomes, (ii) integrate multi-omics technologies and microbial cultures, (iii) elucidate environmental variables and climate change, and (iv) cope with agro-economy, plant production, and food security systems. The ability of phytomicrobiome *vs*. phytobiome that largely cover micro and macro organisms and provide a secondary sanctuary of microbial species to transmit and translocate from one compartment into another during symbiosis is essential to consider (Leach et al., 2017). There is also a greater need to connect the loops of cross-kingdom phytomicrobiome diversity and function with plants, especially during changing environmental conditions. Further, a growing need exists to associate synthetic communities that could augment crop stress performance and nutrient cycling functionalities. Bioinoculums of the native or synthetic microbiome and its diversity across different crops can help establish more significant benefits of eco-friendlier approaches to cope with plant stress tolerance. However, using the reductionism approach, one must maintain a nature-friendlier and native stress-fit microbiome to improve plant growth, development, and yields during extreme heat, drought, and salinity-related stress conditions. Large-scale modified SynComs can exponentially change the natural microbiome diversity associated with plants. Hence, utilizing specific crops through a natural nutrient management system that can improve CO_2_ capture and storage (CCS) will be an essential strategy to overcome the impacts of climate change (Bajaj and Thakur, 2022; Mukherjee, 2022). Recent advances in functional genomics, genome editing technologies, and metabolomics can help discover new genes and pathways adapted by core-microbiome players that could be highly beneficial for identifying plant growth-promoting activities such as biocontrol, biofertilization, and biostimulation. Utilizing network modeling, artificial intelligence, and the internet-of-things based approaches can solve several bottleneck approaches in large-scale field-level studies.

## Author contributions

The author confirms being the sole contributor of this work and has approved it for publication.
